# Impact of post-transplant cyclophosphamide with bendamustine on immune reconstitution in young patients undergoing T-cell replete haploidentical bone marrow transplantation: results from a phase Ia/Ib clinical trial

**DOI:** 10.3389/fimmu.2025.1568862

**Published:** 2025-04-09

**Authors:** Forrest L. Baker, Jessica Stokes, Megan J. Cracchiolo, Dan Davini, Richard J. Simpson, Emmanuel Katsanis

**Affiliations:** ^1^ School of Nutritional Sciences and Wellness, University of Arizona, Tucson, AZ, United States; ^2^ Department of Pediatrics, University of Arizona, Tucson, AZ, United States; ^3^ The University of Arizona Cancer Center, Tucson, AZ, United States; ^4^ Department of Immunobiology, University of Arizona, Tucson, AZ, United States; ^5^ Banner University Medical Center, Tucson, AZ, United States

**Keywords:** bendamustine, cyclophosphamide, hematopoietic cell transplantation, TCR-β sequencing, T-cells, cytomegalovirus

## Abstract

**Introduction:**

Post-transplant cyclophosphamide (PT-CY) has been pivotal in controlling graft-versus-host disease (GvHD) following T-cell-replete haploidentical bone marrow transplantation (haplo-BMT). However, the widely adopted regimen is associated with high relapse rates, particularly in patients without GvHD. Our preclinical studies indicate that pre- or post-transplant bendamustine (PT-BEN) may reduce GvHD, enhance graft-versus-leukemia (GvL) effects, and induce significant alterations in the proportion, phenotype, and function of various immune cell subsets.

**Methods:**

We initiated a Phase Ia/Ib, single-center trial with a standard 3 + 3 dose-escalation design, sequentially replacing post-transplant (PT)-CY with BEN (PT-CY/BEN). Multi-parameter flow cytometry and TCR β sequencing of genomic DNA was performed on isolated PBMCs on PT days +30, +60, +100, +180, and +365.

**Results:**

Overall, the PT-CY/BEN (n=14) regimen was associated with earlier neutrophil and platelet engraftment, reduced transfusion requirements, and comparable clinical outcomes to PT-CY (n=10), including survival and relapse rates. PT-CY/BEN patients exhibited distinct immune reconstitution patterns, characterized by earlier CD4+ T-cell recovery, impaired CD8+ T-cell engraftment, and reduced NK-cell counts. Notably there were no significant changes in B-cells, Tregs, or MDSCs. Enhanced T-cell repertoire diversity in the PT-CY/BEN cohort was associated with improved CMV control.

**Conclusion:**

Our Phase Ia findings demonstrate the well-tolerability of PT-CY/BEN and its association with early engraftment, a more diverse T-cell repertoire, and earlier CD4+ T-cell reconstitution. Future studies are warranted to confirm our findings and investigate potential additional benefits of PT-CY/BEN over PT-CY alone.

## 1 Introduction

Post-transplant cyclophosphamide (PT-CY) significantly reduces the risk of graft-versus-host disease (GvHD), a common complication in T-cell-replete haploidentical bone marrow transplantation (haplo-BMT), and has become the most-widely adopted approach in haplo-BMT (1–3). However, PT-CY is associated with high relapse rates, particularly following reduced intensity conditioning (RIC) (4). Additional complications, such as cytomegalovirus (CMV) reactivation and human polyomavirus 1 (BK) virus-associated cystitis are common, while other complications, such as cardiotoxicity, are rare and less frequent (5–7).

Bendamustine (BEN), a bifunctional alkylating agent and purine analogue, is being investigated as an alternative to CY due to its distinct mechanisms of action in GvHD prophylaxis (8, 9). CY primarily works by selectively depleting alloreactive T-cells while sparing regulatory T-cells, due to their high expression of aldehyde dehydrogenase (10–13). While both agents activate DNA repair pathways, BEN elicits more extensive DNA damage may lead to greater activation of double-strand break repair mechanisms, potentially influencing its efficacy in transplantation settings (14, 15). Importantly, our published preclinical research in mice supported the replacement of CY with BEN by demonstrating that PT-BEN was equally effective in preventing early GvHD and protecting against late GvHD, while enabling superior GvL when compared to PT-CY (16). Since our initial report in 2016 and using various preclinical models, our laboratory has delineated several immunomodulatory properties of BEN elucidating its role in modulating GvHD and GvL (16–22). Irrespective of whether BEN is given pre- or post-transplant, we have reliably observed decreased GvHD, increased GvL, and significant changes in the proportion and phenotype of multiple immune cell types (19). These have included effects of BEN on MDSCs and DC subsets (17, 22) and generation of tolerant T-cells with a striking absence of GvHD, while preserving T-cell dependent GvL (18). Additionally, our *in vitro* studies have revealed that BEN increases the suppressive functions of MDSCs, skews DC generation towards cDC1s, promotes DC Flt3 expression, increases B-cell production of IL-10, inhibits STAT3 phosphorylation, and suppresses B- and T-cell proliferation (16–22).

Based on our preclinical findings, we initiated a Phase Ia clinical trial (NCT02996773) to progressively substitute PT-CY with PT-BEN and determine the maximum tolerated dose. Clinically, we observed a progressive improvement in trilineage engraftment and a reduction in transfusion requirements as PT-BEN doses increased and PT-CY doses decreased (23, 24). Notably, the PT-CY/BEN regimen demonstrated promising trends toward significant improvements in chronic GvHD outcomes (cGvHD) and GvHD-free survival (GRFS) compared to PT-CY alone (23, 24). Additionally, there was a trend toward reduced CMV viremia in patients receiving the PT-CY/BEN regimen (23, 24). However, the effects of PT-CY/BEN on immune reconstitution have yet to be fully elucidated.

Immune reconstitution following BMT involves restoring the immune system's capacity to combat infections, prevent disease relapse, and mitigate the risk of secondary malignancies (25). Achieving effective immune reconstitution is critical to minimizing post-transplant complications such as GvHD while promoting GvL effects (12). PT-CY selectively eliminates alloreactive T-cells, curbing their proliferation and impairing the function of any surviving alloreactive T-cells (13). This process fosters the preferential recovery of regulatory T-cells (Tregs), which play a crucial role in GvHD prevention (13). While PT-CY is associated with delayed reconstitution of CD4+ T-cells, it facilitates earlier B-cell recovery compared to conventional GvHD prophylaxis strategies (26).

In the current study, we aimed to characterize the effects of PT-CY/BEN on immune reconstitution after T-cell-replete haplo-BMT. As we have previously reported the immunomodulatory effects of PT-BEN *in vitro* and *in vivo*, we anticipated that the patients receiving PT-CY/BEN compared to PT-CY alone would demonstrate differences in immune reconstitution.

## 2 Methods

### 2.1 Phase Ia/1b clinical trial

Patients (n=24) with hematologic malignancies who underwent myeloablative conditioned (MAC) T-replete haplo-BMT between January 2017 and August 2022, and were enrolled on a Phase Ia/Ib single-institution trial (NCT02996773), were included in this analysis (23, 24, 27, 28). Eligible patients were between the ages of 0 and 44, lacked a matched related donor, met the organ function criteria for MAC, and had no evidence of active untreated infection. According to diagnosis and disease characteristics, patients received either fractionated total body irradiation (TBI) followed by fludarabine (FLU) or busulfan (BU), fludarabine (FLU), and melphalan (MEL) combination, as previously reported (**Table 1**) (29, 30). All patients received bone marrow grafts on day 0. The Phase Ia/Ib trial was approved by our institutional review board (IRB) and all patients were provided and signed a written informed consent. The study was conducted in accordance with the Declaration of Helsinki.

**Table 1:** Patient, disease and transplant characteristics.

	PT-CY/BEN	PT-CY	*P=*
N=	14	10	
**Age**, median yr, (range)	20.4 (9-42)	26.5 (11-44)	*0.11*
**Male**, n (%)	9 (64)	6 (60)	*0.99*
Race/Ethnicity, n (%)			*0.22*
African American	1 (7)	3 (30)	
White Hispanic	7 (50)	4 (40)	
White non-Hispanic	6 (43)	3 (30)	
Diagnosis, n (%)			*0.58*
ALL	6 (43)	4 (40)	
AML/MDS	3 (21)	3 (30)	
AUL/MLL	1 (7)	1 (10)	
CML	1 (7)	2 (20)	
NHL	3 (21)	0 (0)	
Clinical Service, n (%)			*0.20*
Pediatric	11 (79)	5 (50)	
Adult	3 (23)	5 (50)	
Pretransplant Status, n (%)			*0.59*
CR1	5 (36)	4 (40)	
CR2	3 (21)	3 (30)	
>CR2	3 (21)	1 (10)	
other	3 (21)	2 (20)	
Pretransplant Status, n (%)			
Prior HCT	0 (0)	1 (10)	*0.42*
Prior CAR-T	1 (7)	0 (0)	*0.99*
Disease risk index, n (%)			*0.55*
Low	1 (7)	2 (20)	
Intermediate	10 (71)	6 (60)	
High	3 (21)	2 (20)	
Lansky/Karnofsky PS, n (%)			*0.99*
90-100	10 (71)	7 (70)	
≤80	4 (29)	3 (30)	
HCT Comorbidity index, n (%)			*0.20*
≤2	11 (79)	5 (50)	
≥3	3 (21)	5 (50)	
Conditioning, n (%)			*0.70*
TBI-FLU	7 (50)	4 (40)	
BU-FLU-MEL	7 (50)	6 (60)	
**Donor age**, median yr, (range)	27.9 (15-57)	35.3 (20-62)	*0.51*
Sex mismatch, n (%)			*0.40*
Female ➔ Male	3 (21)	3 (30)	
Male ➔ Female	1 (7)	2 (20)	
None	10(71)	5 (50)	
HLA Match, n (%)			*0.04*
5/10	12 (86)	4 (40)	
6/10	1 (7)	4 (40)	
7/10	1 (7)	1 (10)	
8/10	0 (0)	1 (10)	
RBC incompatibility, n (%)			*0.50*
Major	5 (36)	3 (30)	
Minor	2 (14)	0 (0)	
None	7 (50)	7 (70)	

CY, cyclophosphamide; BEN, bendamustine; ALL, acute lymphoblastic leukemia; AML, acute myeloid leukemia; MDS, myelodysplastic syndrome; AUL/MLL, acute undifferentiated or mixed lineage leukemia; CML, chronic myeloid leukemia; NHL, non-Hodgkin lymphoma; HD, Hodgkin’s disease; CR, complete remission; HCT, hematopoietic cell transplant; CAR-T, chimeric antigen receptor T-cells, TBI, total body irradiation; FLU, fludarabine; BU, busulfan; MEL, melphalan.

The phase Ia was a standard 3 + 3 dose escalation design with the first three cohorts receiving on day +4 PT-CY (mg/kg)/PT-BEN (mg/m^2^): 40/20, 20/60, and 0/90 (23, 27). All patients received PT-CY 50 mg/kg on day +3. Cohort 4 patients received PT-CY/BEN 40/20 on day +3 and only PT-BEN 90 mg/m^2^ on day +4. Cohort 3 dosing (day +3 CY 50 mg/kg and day +4 BEN 90 mg/m^2^) was deemed the maximal tolerated dose (MTD) as we transitioned to Phase Ib, with two additional patients receiving PT-CY on day +3 and PT-BEN on day +4. A total of 14 patients consented to participate in the study and received PT-CY/BEN. Additionally, 10 patients undergoing haplo-BMT with the same conditioning regimens opted not to receive PT-BEN, but consented to be enrolled as PT-CY controls for clinical endpoint evaluations and immune reconstitution analyses. All patients received mycophenolate mofetil on day +5 through day +28 and tacrolimus starting on day +5. In the absence of GvHD, tacrolimus was tapered starting day +70 to +90 and discontinued by day +120 to +180. GvHD was graded according to the consensus criteria for grading acute and chronic GvHD (31, 32).

### 2.2 Engraftment and donor chimerism and immune reconstitution monitoring

Granulocyte stimulating factor (G-CSF) was started on day +5 at 5 μg/kg/day until an absolute neutrophil count (ANC) of 2.5 x 10^9^/L was achieved for three consecutive days. Day of myeloid engraftment was defined as the first of three consecutive days with an ANC of >0.5 x 10^9^/L. Day of platelet engraftment was considered the first of three consecutive days with platelet counts of >20 x 10^9^/L with no platelet transfusions administered in the previous 7 days. Donor chimerism and immune reconstitution studies were performed on PT days +30±5, +60±7, +100±10, +180±15, +365±30.

### 2.3 Immune reconstitution

To analyze peripheral immune cell subsets using flow cytometry, we collected venous blood into EDTA-anticoagulated tubes and peripheral blood mononuclear cells (PBMCs) were isolated using a density gradient (Ficoll-Paque Plus). PBMCs were Fc blocked and then incubated with fluorochrome-labeled monoclonal antibody cocktails (**Supplementary Table 1**). Specifically, PBMCs were incubated with antibodies for 30 minutes in the dark at room temperature. For intracellular staining of FoxP3, labeled PBMCs were fixed (30 minutes) and permeabilized (Fixation/Permeabilization; eBioscience, San Diego, CA), and stained with anti-FoxP3 for 30 mins. Immunophenotyping data were collected with an LSRFortessa cell analyzer (BD Biosciences) and analyzed using FlowJo (BD Life Sciences). The percentages and absolute counts were calculated for T-cells (CD3^+^), CD4+ T-cells (CD3^+^/CD4^+^), CD8+ T-cells (CD3^+^/CD8^+^), gamma-delta T-cells (CD3^+^/γδTCR^+^), NKT-cells, (CD1d^+^/CD3^+^/CD56^+^), NK-cells (CD3^-^CD56^+^), Naïve (CD4^+^/CD25^+^/CD127^-^/CD45RA^+^/FoxP3^+/low^) and Effector (CD4^+^/CD25^+^/CD127^-^/CD45RA^-^/FoxP3^+^) Tregs, B-Cells (CD19^+^), Monocytes (CD14^+^/HLA-DR^+^), Granulocytic (CD11b^+^/CD14^-^/CD15^+^/CD33^+^/HLA-DR^-/low^) and Monocytic (CD11b^+^/CD14^+^/CD15^+low^/CD33^+^/HLA-DR^-/low^) MDSCs, and Dendritic Cells (Lin^-^/CD11c^+^). For CD4+ and CD8+ T-cells the following differentiation subsets were calculated, recent thymic emigrant (RTE) naïve (CCR7^+^/CD45RA^+^/CD95^-^/CD31^+^), naïve (CCR7^+^/CD45RA^+^/CD95^-^/CD31^-^), stem cell memory (SCM) (CCR7^+^/CD45RA^+^/CD95^+^), central memory (CM) (CCR7^+^/CD45RA^-^), effector memory (EM) (CCR7^-^/CD45RA^-^), terminally differentiated CD45RA+ effector memory (EMRA) (CCR7^-^/CD45RA^+^). NK-cells were further differentiated into immature (CD3^-^/CD14^-^/CD56^bright^/CD16^-^) and mature cytotoxic (CD3^-^/CD14^-^/CD56^dim^/CD16^+^) subsets. B cell memory status was classified as naive mature (CD19^+^/CD27^-^/CD24^int^/CD38^int^), transitional (CD19^+^/CD27^-^/CD24^++^/CD38^++^), memory (CD19^+^/CD27^+^/CD24^+^/ CD38^-/int^). In all panels, doublets were excluded, and single-color controls were included.

### 2.4 DNA isolation and TCR sequencing

Genomic DNA (gDNA) was extracted using the DNeasy Blood Extraction Kit (Qiagen, Germantown, MD) from freshly isolated PBMCs. Genomic DNA was sent to Adaptive Biotechnologies (Seattle, WA) for TCR-β sequencing (*n*=21) and data analysis was performed using the ImmunoSEQ platform. CMV specific clones were determined using ImmunsoSEQ’s CMV classifier and all data was analyzed on the platform between 2019-2022.

### 2.5 Statistical analysis

All statistical analysis was done using GraphPad Prism 9 software (La Jolla, CA). Comparisons of patient characteristics and outcome variables between the PT-CY and PT-CY/BEN groups were performed using Fisher’s Exact Tests for categorical variables and Mann-Whitney/Wilcoxon Rank Sum Tests for continuous variables. Repeated measures one-way analysis of variance (ANOVA) with Tukey’s *post-hoc* tests were performed to determine immune reconstitution differences between PT-CY and PT-CY/BEN groups at each time point. Data were log-transformed if checks for normality failed. Chi-square analyses were used to determine differences between CD4+ T-cell engraftment (>50 cells/μL) at each time point. Time to event endpoints were estimated using cumulative incidence curves and Kaplan-Meier curves, with comparisons using log-rank tests.

## 3 Results

### 3.1 Patient, disease and transplant characteristics

The trial included 14 patients in the experimental arm and 10 controls for the immune reconstitution studies. Clinical outcomes for 13 of the 14 patients were previously reported and compared with 31 synchronous PT-CY controls (23). However, only 10 of these controls consented to participate in the serial immune reconstitution studies. Therefore, this analysis provides a more concise summary of clinical outcomes specifically for the controls who underwent immune reconstitution evaluation, in comparison to all patients in the experimental arm, and includes an additional 33 months of follow-up for patients in both study arms. Patient and transplant characteristics are summarized in **Table 1**. Baseline characteristics such as age, sex, race/ethnicity, diagnosis, clinical service, pre-transplant remission status, disease risk index, performance status, comorbidity index, conditioning regimen, donor age, sex mismatch, and HLA match were comparable between the PT-CY/BEN and PT-CY groups, with the exception of HLA antigen match, which favored the control group (**Table 1**).

### 3.2 Engraftment and chimerism

**Figure 1:**
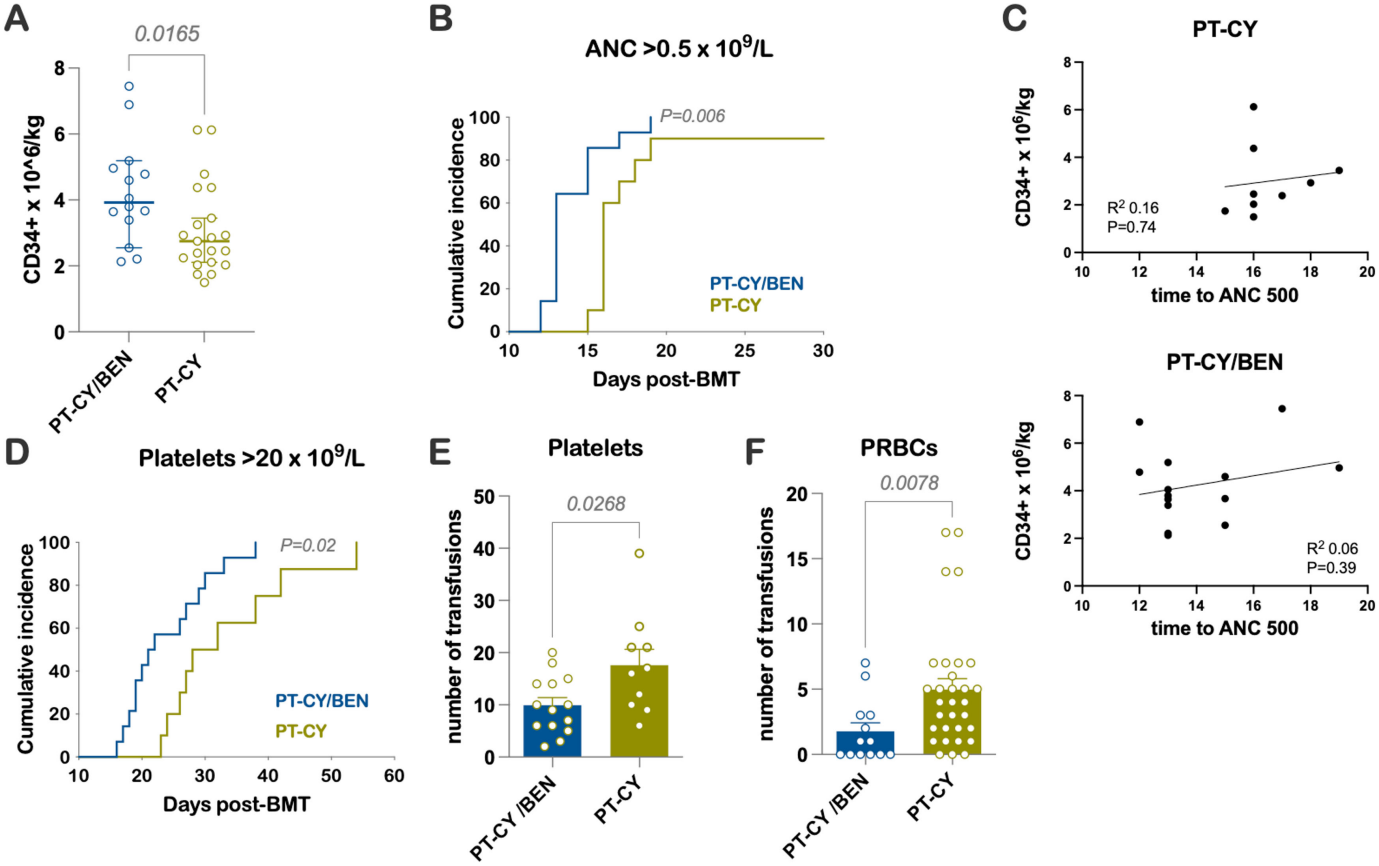
Early Engraftment Kinetics and Transfusions **(A)** Number of CD34^+^ cells × 10^6^/kg infused. **(B)** Time to an ANC of 0.5 × 10^9^/L. **(C)** Correlation between the number of CD34+ cells infused and time to myeloid engraftment. **(D)** Time to a platelet count of 20 × 10^9^/L. Units of **(E)** platelets and **(F)** packed RBCs (PRBCs) transfused.

PT-CY/BEN patients received a median of 3.9 x10^6^/kg CD34^+^ cells compared to 2.8 x10^6^/kg in the PT-CY group (*P=0.02*) (**Figure 1A**). Patients treated with PT-CY/BEN had earlier trilineage engraftment, with a median time to an absolute neutrophil count (ANC) of 0.5 x 10^9^/L of 13 days compared to 16 days in those receiving PT-CY (*P=0.006)* (**Figure 1B**). However, we found no correlation between the number of CD34+ cells infused and time to myeloid engraftment (**Figure 1C**). Similarly, PT-CY/BEN patients demonstrated earlier platelet engraftment, with a median of 22 days compared to 30 days in PT-CY patients (*P=0.02)* (**Figure 1D**). Consequently, PT-CY/BEN patients required fewer platelet *(P=0.03)* and red blood cell transfusions *(P=0.008)* (**Figures 1E, F**). Trilineage engraftment was observed in all patients, except for two who experienced graft failure—one from each group. These patients were successfully salvaged with a second haploidentical hematopoietic cell transplant (HCT) performed 30 and 46 days later, utilizing a single-day conditioning regimen (33). Due to the graft failure and subsequent transplant, these two patients were excluded from the immune reconstitution studies.

### 3.3 Clinical outcomes

**Figure 2:**
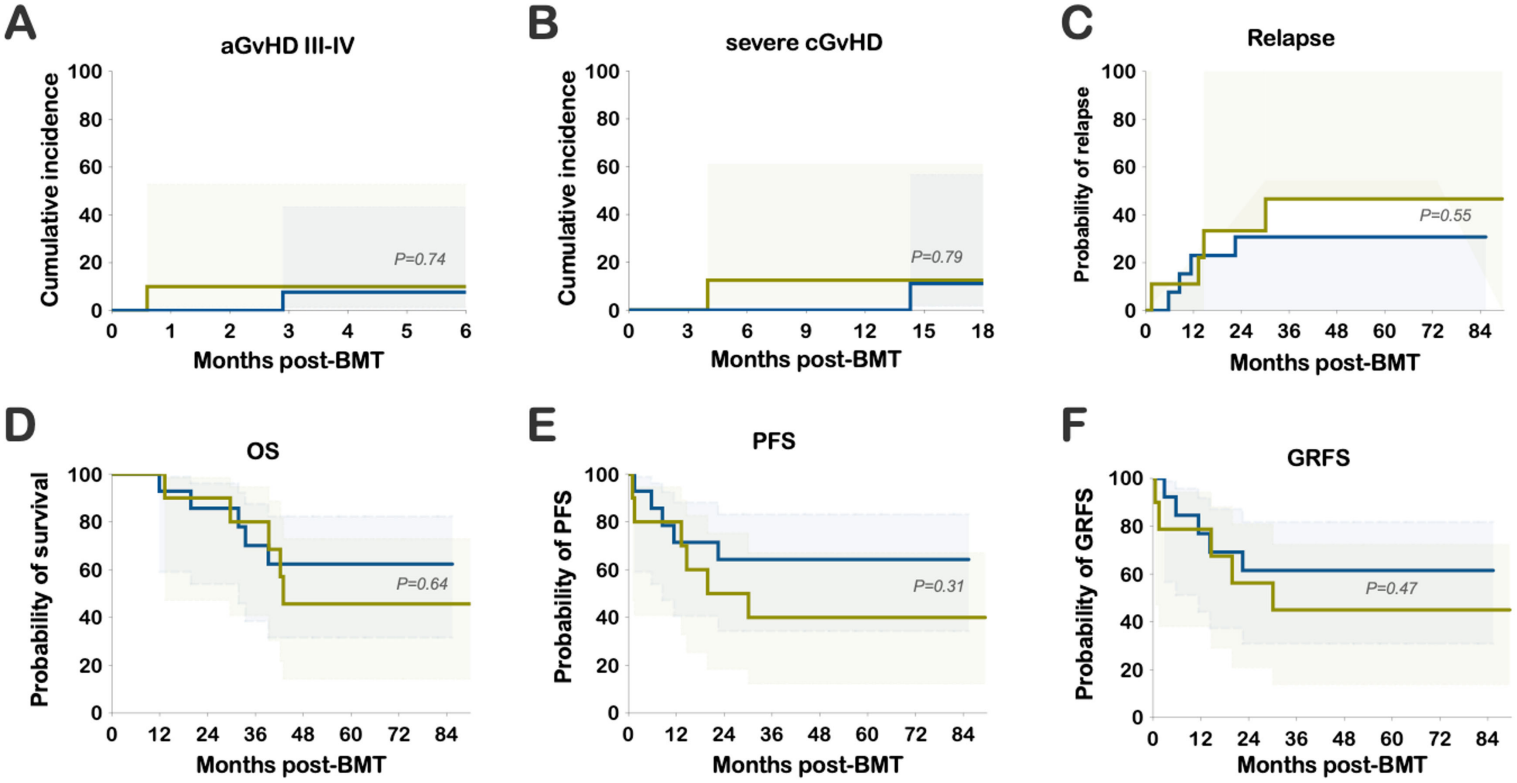
Clinical Outcomes: Cumulative incidence of **(A)** grade III-IV acute GvHD (aGvHD) and **(B)** severe chronic GvHD (cGvHD). Probability of **(C)** relapse, **(D)** OS, **(E)** PFS, and **(F)** GRFS.

The median follow-up was 66 months (range 29 to 90). The cumulative incidence of grades III-IV aGvHD was low, observed at 7.7% in the PT-CY/BEN group compared to 10% in the PT-CY-alone group (*P=ns*) (**Figure 2A**). Similarly, the cumulative incidence of severe cGvHD requiring systemic treatment was comparable, at 11.1% in PT-CY/BEN patients versus 12.5% in PT-CY controls (*P=ns*) (**Figure 2B**). Relapse rates were not significantly different, with 30.8% of patients in the PT-CY/BEN group experiencing relapse compared to 46.7% in the PT-CY group (*P*=*ns*) (**Figure 2C**). The probabilities of overall survival (OS), event-free survival (EFS), and GvHD-free relapse-free survival (GRFS) at 84 months were similar between groups, at 62.3% versus 45.7%, 64.3% versus 40%, and 61.5% versus 45%, respectively, for PT-CY/BEN and PT-CY (*P=ns*) (**Figures 2D–F**).

**Figure 3:**
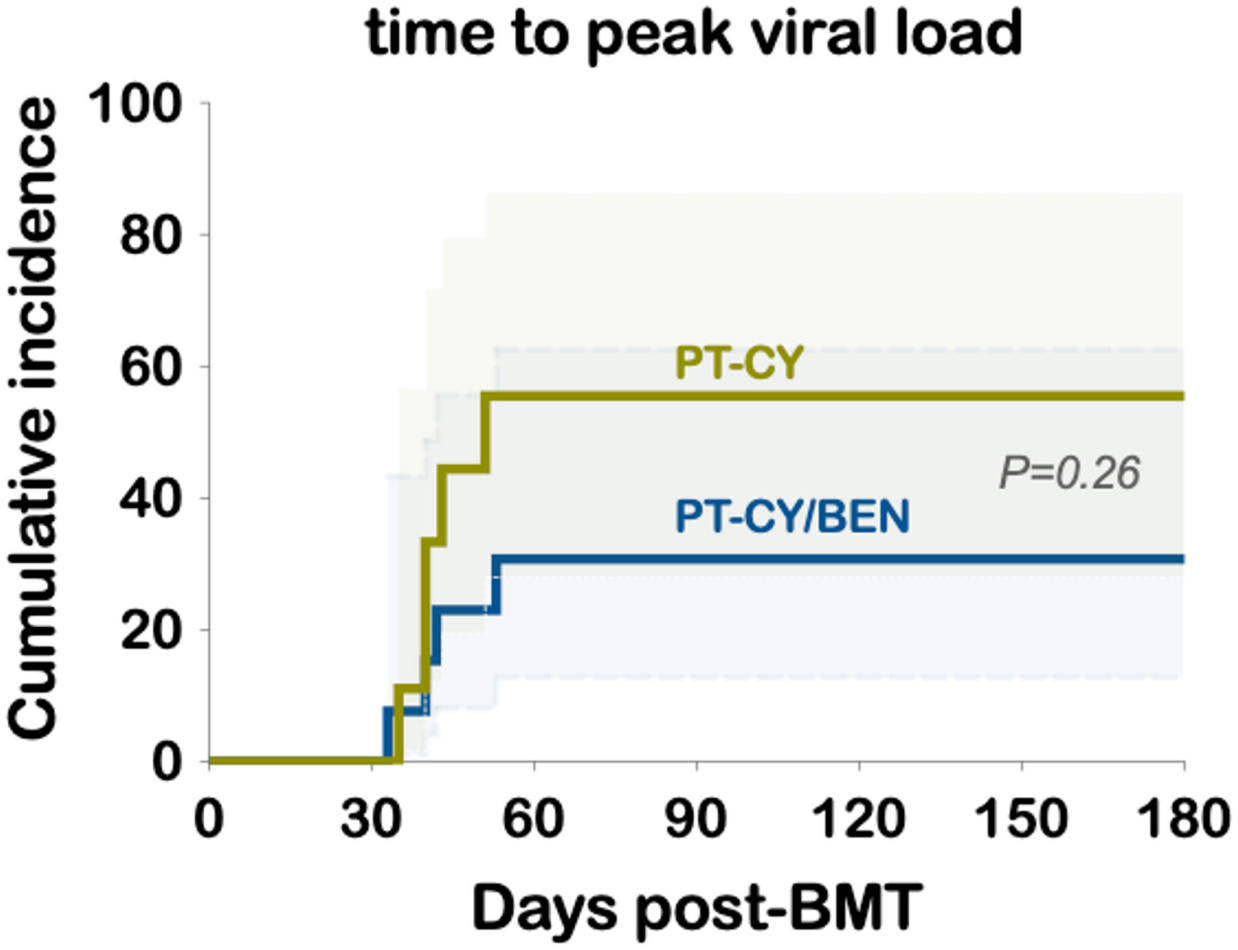
Cumulative incidence of CMV viremia and time to peak viral load.

The cumulative incidence of CMV reactivation was 30.8% in the PT-CY/BEN group and 55.6% in PT-CY controls (*P=0.26*) (**Figure 3**). Notably, 3 of 14 patients in the PT-CY/BEN group were CMV seronegative along with their donors, compared to 1 of 10 in the PT-CY group. Additionally, only one control patient received letermovir prophylaxis and none in the PT-CY/BEN group. BK viruria was detected in 4 PT-CY patients (40%) with >5x108 viral copies/mL and symptoms of BK hemorrhagic cystitis, compared with 3 PT-CY/BEN patients (21.4%) (*P=0.393*; *data not shown*). None of the patients in either group had clinically significant reactivation of EBV, HHV-6, or adenovirus warranting therapeutic intervention.

### 3.4 PT-CY/BEN facilitates an earlier CD4+ T-cell engraftment but impairs CD8+ T-cell engraftment at one-year post-transplant

**Figure 4:**
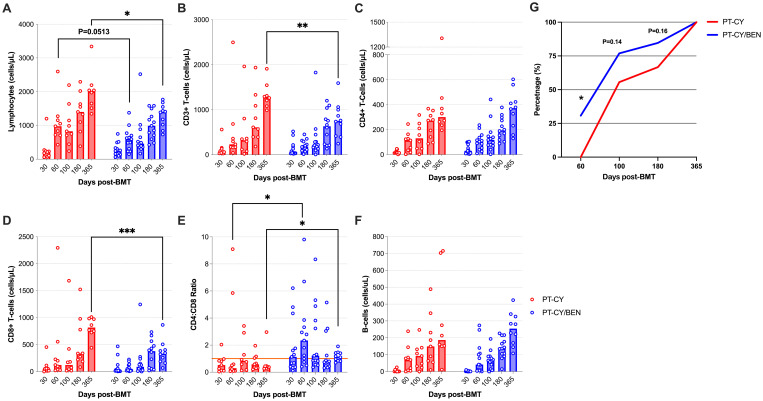
Reconstitution of major lymphocyte populations after T-cell replete haplo-BMT using post-transplant cyclophosphamide (PT-CY, red line) compared to post-transplant cyclophosphamide/bendamustine (PT-CY/BEN, blue line) GvHD prophylaxis. Reconstitution of **(A)** Lymphocytes, **(B)** CD3+ T-cells, **(C)** CD4+ T-cells, **(D)** CD8+ T-cells, **(E)** CD4:CD8 ratio, and **(F)** B-Cells. **(G)** Percentage of the patients in each cohort that achieved a CD4+ T-cell count of 50 cells/μL. Bars represent median cell counts per microliter at each time point. Statistically significant differences are noted in each figure (***P < 0.001; **P < 0.01; *P < 0.05).

We comprehensively analyzed the T-cell compartments in patients receiving PT-CY/BEN (*n*=13) versus PT-CY (*n*=9) alone using multicolor flow cytometry. Due to the small sample size of each PT-CY/BEN cohort (*n*=3), all PT-CY/BEN cohorts were combined into a single group to evaluate the impact of substituting PT-CY with PT-BEN on immune reconstitution. Overall, the lymphocyte counts trended lower at two months (*P=0.051*) and were significantly lower one-year (*P=0.014*) post-BMT in patients receiving PT-CY/BEN (**Figure 4A**). We also observed reduced total CD3+ T-cell counts at one-year (*P=0.0035*) post-transplant in the PT-CY/BEN patients (**Figure 4B**). While the reconstitution of CD4+ T-cells did not appear to be significantly altered over the first year post-BMT between groups, patients in the PT-CY/BEN group achieved earlier reconstitution of CD4+ T-cells to levels >50 cells/µL compared to the PT-CY group (*P=0.03*) (**Figures 4C, G**). Early CD4+ T cell immune reconstitution defined as >50 cells/µL on two consecutive measures within 100 days of HCT has been shown to be a predictor of improved survival post-BMT (34).The reduction in total CD3+ T-cells was driven by a marked decrease in CD8+ T-cell reconstitution at one-year post-transplant (*P<0.0001*) (**Figure 4D**). This resulted in an elevated CD4:CD8 ratio which was significantly higher than PT-CY controls at the two month (*P=0.041*) and one-year mark (*P*=0.014) (**Figure 4E**). Finally, the reconstitution of B-cells was not affected by PT-CY/BEN (**Figure 4F**).

### 3.5 PT-CY/BEN is associated with minor changes in CD8+ T-cell differentiation one-year post-transplant, but it does not affect the CD4+ T-cell or B-cell compartment

**Figure 5:**
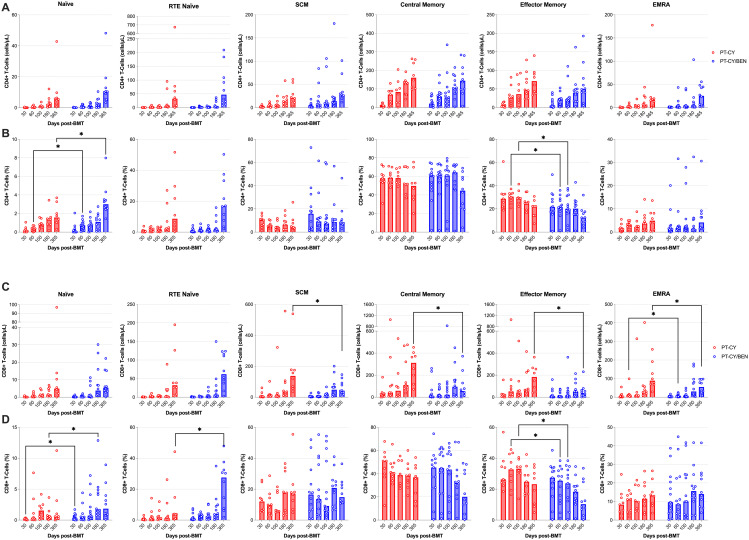
Reconstitution of T-cell memory populations after T-cell replete haplo-BMT. Reconstitution of CD4+ **(A, B)** and CD8+ **(C, D)** T-cells Naïve, RTE Naïve, SCM, CM, EM, and EMRA subsets, represented as absolute counts (cells/μL) and frequency (%). Post-transplant cyclophosphamide (PT-CY, red line) compared to post-transplant cyclophosphamide/bendamustine (PT-CY/BEN, blue line) GvHD prophylaxis. Bars represent median cell counts per microliter or percentage at each time point. Statistically significant differences are noted in each figure (*P < 0.05).

We next evaluated whether PT-CY/BEN impacts the composition of the CD4+ and CD8+ T-cell compartments, with a focus on memory status. Within the CD4+ T-cell compartment no significant differences were observed in the counts of naïve, recent thymic emigrant (RTE) naïve, central memory (CM), stem cell memory (SCM), effector memory (EM), or effector memory RA (EMRA) CD4+ T-cells between PT-CY/BEN and PT-CY patients at any time point (**Figure 5A**). However, the proportion of naïve CD4+ T-cells 60-days (*P=0.036*) and one-year (*P=0.036*), as well as, the proportion of EM CD4+ T-cells 60-days (*P=0.013*) and 100-days (*P=0.031*) post-transplant were significantly lower in the PT-CY/BEN patients (**Figure 5B**). No differences were observed in the proportions of RTE naïve, SCM, CM, or EMRA CD4+ T-cells at any timepoint. In contrast, within the CD8+ T-cell compartment, PT-CY/BEN patients exhibited a significant reduction in SCM (*P=0.031*), CM (*P=0.012*), EM (*P=0.021*), and EMRA (*P=0.042*) CD8+ T-cell counts in PT-CY/BEN patients at one-year post-transplant (**Figure 5C**). At 60-days post-transplant, EMRA CD8+ T-cell counts were significantly reduced in PT-CY/BEN patients compared to PT-CY patients (*P=0.043*) (**Figure 5C**). No differences were observed in naïve or RTE naïve CD8+ T-cell counts at any timepoint. The proportion of naïve CD8+ T-cells one-month (*P=0.033*) and 180-days (*P=0.048*), as well as the proportion of RTE naïve CD8+ T-cells one-year (*P=0.031*) and 100-days (*P=0.031*) post-transplant were significantly elevated in the PT-CY/BEN patients (**Figure 5D**). Alternatively, the proportion of EM CD8+ T-cells 60-days (*P=0.049*) and 100-days (*P=0.036*) post-transplant were significantly lower in the PT-CY/BEN patients (**Figure 5D**). No differences were observed in the proportions of SCM, CM, or EMRA CD8+ T-cells at any timepoint. Similarly, there were no significant changes in naïve mature, transitional, or memory B-cell counts between PT-CY/BEN and PT-CY patients at any time point post-transplant (*data not shown*).

### 3.6 NK-cells are reduced in PT-CY/BEN patients late post-transplant but γδ T-cells are unaffected

**Figure 6:**
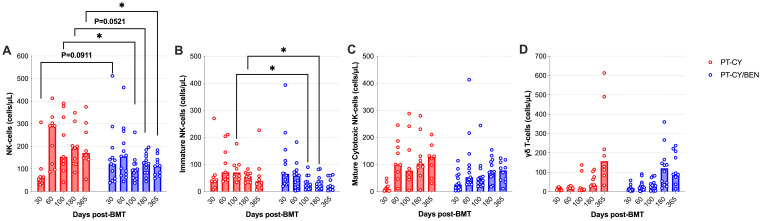
Reconstitution of NK and γδ T-cells populations after T-cell replete haplo-BMT. Reconstitution of **(A)** NK-Cells, **(B)** Immature NK-cells **(C)** Mature Cytotoxic NK-Cells, and **(D)** γδ T-cells. Post-transplant cyclophosphamide (PT-CY, red line) compared to post-transplant cyclophosphamide/bendamustine (PT-CY/BEN, blue line) GvHD prophylaxis. Bars represent median cell counts per microliter at each time point. Statistically significant differences are noted in each figure (*P < 0.05).

We next evaluated if PT-CY/BEN affects the composition of NK-cells and γδ T-cells, given their anti-tumoral and anti-viral importance post-transplant. We observed a significant reduction in NK-cell counts at 100-days (*P=0.039*) and one-year post-transplant (*P=0.047*), as well as a trend towards reduced NK-cell counts 6-months (*P=0.052*) post post-transplant (**Figure 6A**). Moreover, a trend towards increased NK-cells at Day 30 (*P=0.09*) was observed in the PT-CY/BEN cohort (**Figure 6A**). Within the NK-cell compartment, the decrease in total NK-cells was driven by significant decreases among immature NK-cells in PT-CY/BEN patients at 100-days (*P=0.019*) and 6 months (*P=0.022*) post-transplant (**Figure 6B**). However, no significant differences were observed in the counts of mature cytotoxic NK-cells between PT-CY/BEN and PT-CY patients at any time-point (**Figure 6C**). Finally, γδ T-cells counts between PT-CY/BEN and PT-CY patients did not significantly differ at any time-point (**Figure 6D**).

### 3.7 Tregs and MDSCs are unaltered in patients receiving PT-CY/BEN

**Figure 7:**
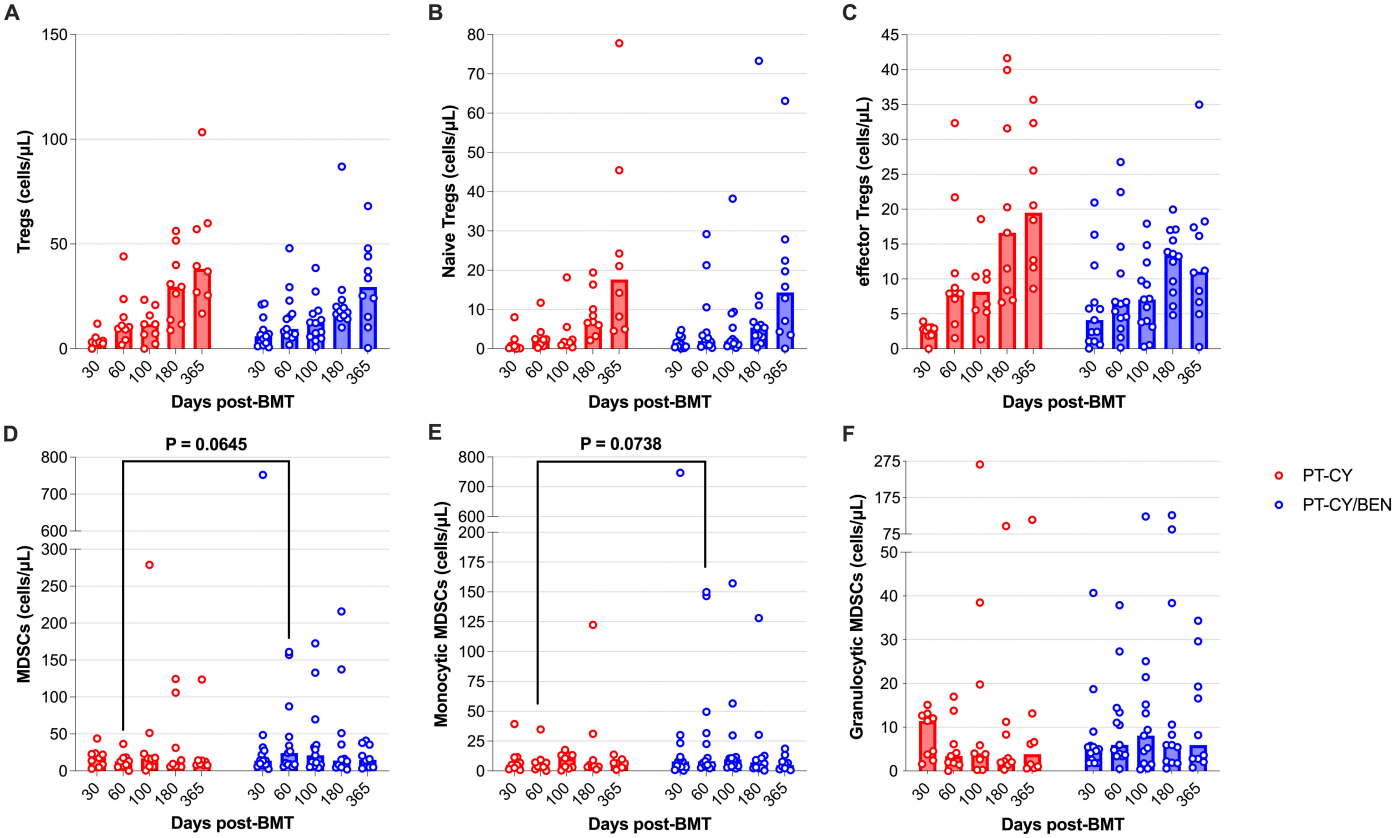
Reconstitution of Tregs and MDSCs populations after T-cell replete haplo-BMT. Reconstitution of **(A)** Tregs, **(B)** Naïve Tregs **(C)** effector Tregs, **(D)** MDSCs, **(E)** Monocytic MDSCs, and **(F)** Granulocytic MDSCs. Post-transplant cyclophosphamide (PT-CY, red line) compared to post-transplant cyclophosphamide/bendamustine (PT-CY/BEN, blue line) GvHD prophylaxis. Bars represent median cell counts per microliter at each time point. Statistically significant differences are noted in each figure.

Given the prominent role MDSCs and Tregs in mitigating GvHD, we assessed whether PT-CY/BEN compared to PT-CY alone affects the composition of MDSC and Treg compartments. Total Tregs counts, as well as naïve and effector T-reg subsets were not significantly different between PT-CY/BEN and PT-CY patients at any time point (**Figures 7A–C**). However, we observed a trend toward higher total MDSC levels (*P=0.065*) two-months post-transplant in PT-CY/BEN patients, driven primarily by a trend towards higher monocytic MDSCs (*P=0.074*) (**Figures 7D, E**). In contrast, granulocytic MDSCs did not differ between the two groups at any time point (**Figure 7F**).

### 3.8 PT-CY/BEN is associated with a more diverse T-cell repertoire, driven by improved CMV control

**Figure 8:**
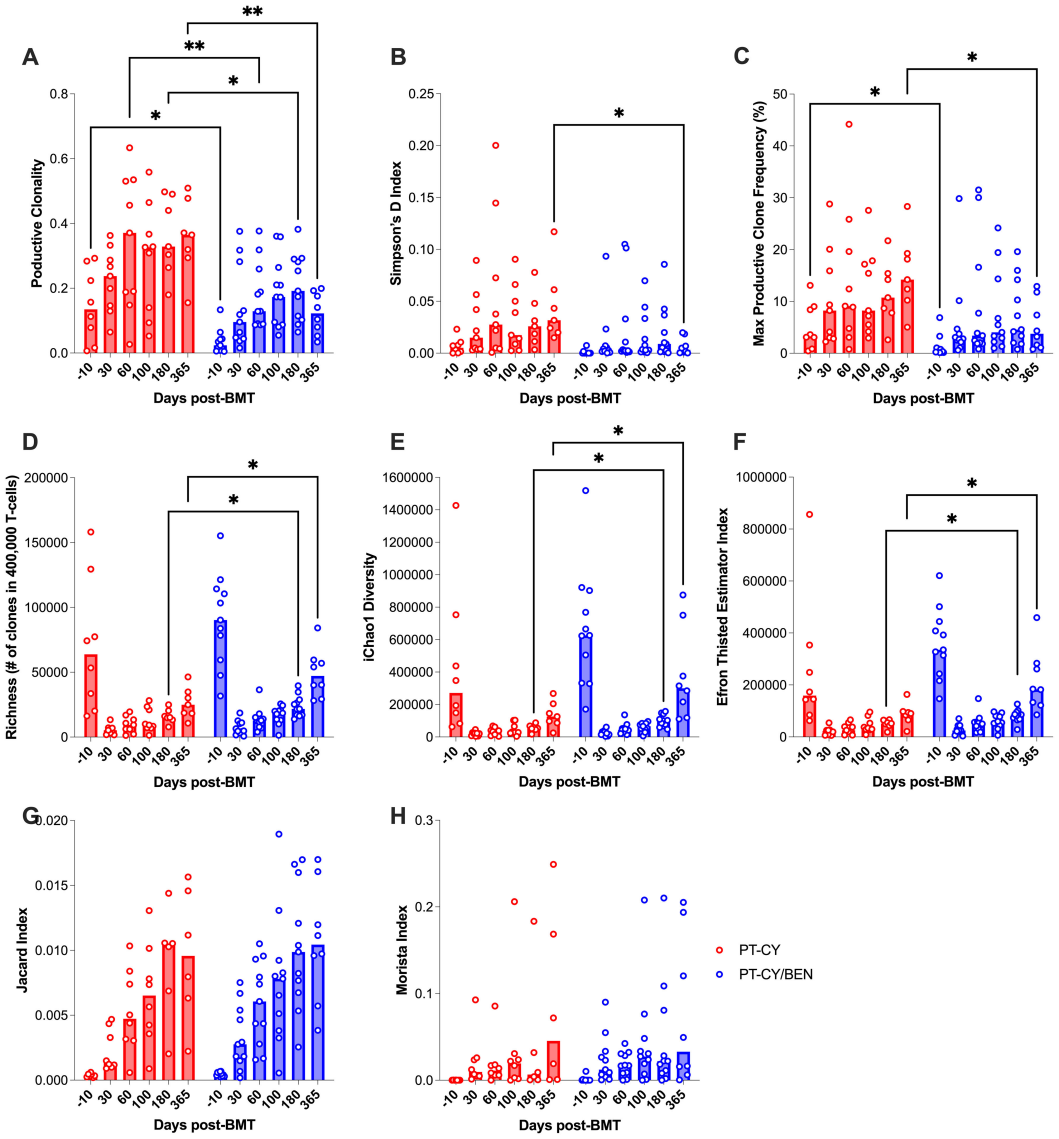
Immunosequencing of the TCR-β chains from PBMCs collected after T-cell replete haplo-BMT. The effect of PT-GvHD prophylaxis on T-cell Diversity, **(A)** Productive Clonality, **(B)** Simpson’s D, **(C)** max productive frequency, **(D)** Richness, **(E)** iChao1 Diversity, and **(F)** Efron Thisted Estimator. The effects of PT-GvHD prophylaxis on donor-patient overlap as measured by the **(G)** Jaccard and **(H)** Morista Index. Post-transplant cyclophosphamide (PT-CY, red) compared to post-transplant cyclophosphamide/bendamustine (PT-CY/BEN, blue) GvHD prophylaxis. Bars represent median TCR-β Diversity metric at each time point. Statistically significant differences are noted in each figure (**P < 0.01; *P < 0.05).

TCR-beta diversity is critical in haploidentical transplantation, as it enhances adaptive immune responses, facilitating pathogen clearance while mitigating the risk of GvHD. To assess the impact of PT-CY/BEN on αβ-T-cell diversity compared to PT-CY alone, we utilized the Adaptive Biotechnologies TCR-β sequencing platform. Using two global measures of T-cell diversity, productive clonality and Simpson’s D index, we observed a more diverse T-cell repertoire in PT-CY/BEN patients (**Figure 8**). Specifically, productive clonality was significantly lower in PT-CY/BEN patients at baseline (*P=0.048*), two months (*P=0.003*), six months (*P=0.039*), and one year (*P=0.003*) post-transplant compared to PT-CY patients (**Figure 8A**). Additionally, PT-CY/BEN was associated with a lower Simpson’s D index at one year (*P=0.03*) and a lower maximum productive clone frequency at baseline (*P=0.046*) and one year (*P=0.013*) post-transplant (**Figures 8B, C**). Richness was also improved in PT-CY/BEN patients at six months (*P=0.038*) and one year (*P=0.014*) post-transplant (**Figure 8D**). This increased richness in clonality was further supported by significant increases in iChao1 (*P=0.022* & *P=0.027*) and Efron-Thisted Estimator (*P=0.027* & *P=0.012*) at six months and one year, respectively (**Figures 8E, F**). PT-CY/BEN had no significant effect on donor-patient repertoire overlap (Jaccard and Morisita Index) at any time point (**Figures 8G, H**).

**Figure 9:**
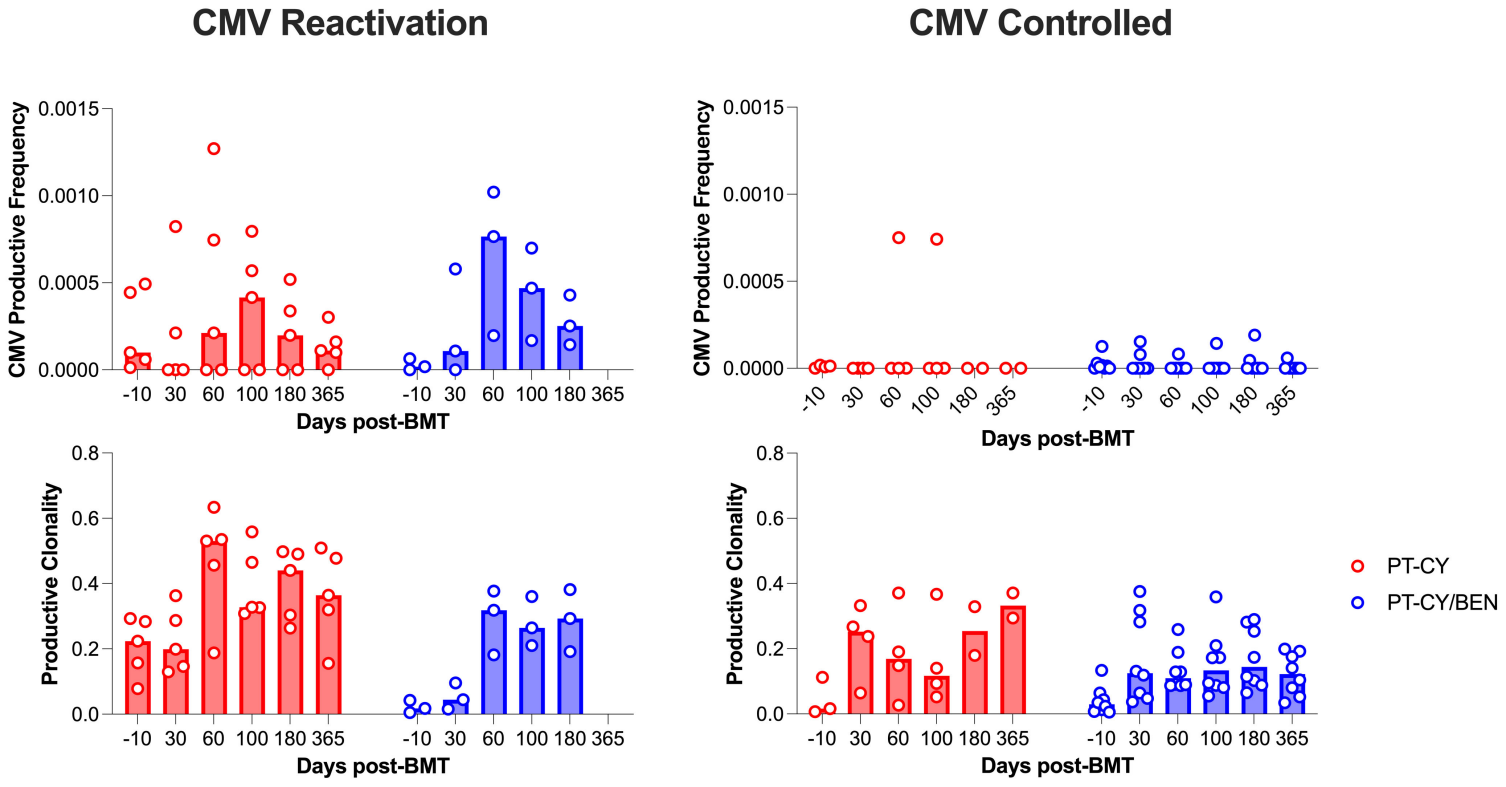
The effect of CMV reactivation on CMV productive frequency and overall productive clonality. Post-transplant cyclophosphamide (PT-CY, red) compared to post-transplant cyclophosphamide/bendamustine (PT-CY/BEN, blue) GvHD prophylaxis. Bars represent median TCR-β Diversity metric at each time point.

Given the impact of CMV infection on the T-cell repertoire, we conducted an exploratory analysis to investigate whether the observed differences in T-cell diversity were associated with CMV infection. To achieve this, we employed the CMV classifier (ImmunoSEQ) to identify CMV-specific clones. Expectedly, we observed that patients (PT-CY/BEN or PT-CY) diagnosed with CMV reactivation developed CMV-specific clones, while patients without a reactivation exhibited minimal to no CMV-specific clones (**Figure 9**). Given the limited number of CMV reactivations, statistical analysis was not feasible. However, it appears that the reduced diversity in the PT-CY patients (characterized by increased productive clonality) within the first 6 months post-BMT was primarily driven by CMV reactivation (**Figure 9**).

## 4 Discussion

T-cell-replete haplo-BMT with PT-CY has become a widely accepted alternative to unrelated donor HCT (1–3). Effective and timely reconstitution of T-cell and NK-cell immunity after haplo-BMT is critical to reducing the risks of GvHD, infections, and relapse, which are the leading causes of morbidity and mortality following haplo-BMT (25, 35). To address relapse risk, strategies such as employing myeloablative conditioning (MAC) regimens and reducing PT-CY doses have been explored (36–38). Reduced PT-CY dosing has been shown in clinical trials to preserve acute GvHD protection while promoting early engraftment and reducing toxicity (39–42). As an alternative approach, we investigated partially replacing PT-CY with PT-BEN. In preclinical murine models, we demonstrated that PT-BEN is as effective as PT-CY in preventing GvHD, while providing superior GvL effects (18). These studies highlighted BEN’s immunomodulatory mechanisms, including its effects on MDSCs, DC subsets, and B-cell and T-cell function, achieving effective GvL with minimal GvHD (16, 17, 19, 21, 22, 43). Building on these findings, we initiated a Phase Ia trial (NCT02996773) to evaluate the feasibility of using PT-BEN in pediatric and young adult patients with hematologic malignancies undergoing haplo-BMT (23, 27).

To investigate the effects of PT-BEN on immune reconstitution, we conducted an in-depth immunophenotypic analysis and TCR-β sequencing in T-cell replete haplo-BMT patients treated with PT-CY or PT-CY/BEN. Our analysis demonstrated that partially replacing PT-CY with PT-BEN accelerated trilineage engraftment, facilitated earlier CD4+ T-cell recovery (>50 cells/µL), increased monocytic MDSC reconstitution, while reducing CD8+ T-cell and NK-cell reconstitution. Additionally, PT-CY/BEN was associated with a more diverse and richer T-cell repertoire, potentially driven by the higher incidence of CMV reactivation in the PT-CY patients. While the small sample size of the two cohorts limits definitive conclusions, these collective findings, together with our previously published clinical data, suggest that PT-CY/BEN may promote early CD4+ T-cell engraftment, potentially induce small alterations in CD8+ T-cell, NK-cell, and MDSC reconstitution, and enhance TCR-β diversity. This may contribute to the observed reduction of chronic GvHD and the improved GRFS outcomes in PT-BEN treated patients when compared to a larger clinical cohort receiving PT-CY alone (23).

In T-cell replete haplo-BMT, early lymphocyte reconstitution is strongly associated with improved survival, primarily due to a reduction in infection-related non-relapse mortality (NRM). T-cell subsets reconstitute through distinct mechanisms following HCT, a process significantly influenced by multiple transplantation and patient-specific factors, including the conditioning regimen, cell source, donor type, recipient and donor age, HLA mismatches, infections, graft manipulation, and GvHD (44). Delayed T-cell reconstitution, often extending beyond two years, is closely linked to increased morbidity and mortality (45). Our findings suggest the kinetics of T-cell reconstitution are largely similar between PT-CY/BEN and PT-CY patients, with some notable differences in CD4+ and CD8+ T-cells. In both cohorts, CD8+ T-cell reconstitution occurred earlier than CD4+ T-cell recovery. However, subtle non-significant differences resulted in higher CD4:CD8 ratios in the PT-CY/BEN patients two months and one-year post-transplant. Importantly, the median CD4:CD8 ratio was less than 1 (0.29-0.84) at all time points in the PT-CY cohort, whereas it was above 1 (0.78-2.4) at all timepoints, except 6 months post-transplant, in the PT-CY/BEN cohort. This elevated CD4:CD8 ratio in PT-CY/BEN patients appears to be driven by lower CD8+ T-cell counts (**Figure 4E**). These findings suggest that PT-CY/BEN suppresses CD8+ T-cell counts, leading to an elevated CD4:CD8 ratio, which may reduce the risk of GvHD. However, we did not observe any significant differences in acute or chronic GvHD between the PT-CY/BEN patients and the smaller PT-CY cohort that had immune reconstitution studies performed in the current report. Alternatively, the elevated CD8+ T-cell and total CD3+ T-cell counts observed one year after transplant in PT-CY patients could be due to a higher incidence of early CMV reactivation. A previous study reported a threefold increase in CD8+ and CD3+ T-cell counts one-year post-HSCT in pediatric patients who experienced early CMV reactivation (46). Additionally, this increased CMV-specific T-cell expansion one year post-transplant is generally driven by EM and EMRA CD8+ T-cell subsets (46) and we observed elevated EM and EMRA CD8+ T-cell counts in the PT-CY cohort at this time point (**Figure 5C**). Importantly, around the time of peak CMV viral load (Days 35–51), the EM and EMRA CD8+ T-cell subsets were proportionally higher in PT-CY patients (**Figure 5D**) (46, 47).

Early CD4^+^ T-cell immune recovery plays a critical role in reducing infection-related mortality and disease recurrence (48, 49). Both the numeric and functional recovery of CD4^+^ T-cells are key predictors of outcomes across various hematopoietic transplant platforms, in pediatric and adult populations (50). While total CD4+ T-cell counts did not differ between the PT-CY/BEN and PT-CY patients at any time point, we did observe an earlier time to CD4+ T-cell engraftment (>50 cells/µL) in the PT-CY/BEN patients (**Figures 4C, D**). This finding is significant, as early CD4+ T-cell engraftment, unlike CD3+ T-cell, CD8+ T-cell, or NK-cell reconstitution, has been associated with improved overall survival and reduced NRM in pediatric recipients of T-replete allogeneic HCT (48, 50, 51). Moreover, the early CD4+ T-cell recovery also aligns with the quicker engraftment of neutrophils (**Figure 1**), as innate immune cell recovery (i.e neutrophils, monocytes, and NK-cells) predicts higher probability of CD4+ T cell reconstitution after pediatric HCT (52). Recent studies have reported that achieving CD4+ T-cell counts of >50 cells/µL on two consecutive assessments within 100 days post-HCT was associated with improved OS and EFS and reduced NRM, but no association with acute GVHD was found (34). In our trial, 30.8% and 76.9% of PT-CY/BEN patients achieved CD4+ T-cell counts on two consecutive assessments by +60 and +100 days compared to 0% and 55.6% of PT-CY patients respectively (**Figure 4G**), suggesting that PT-CY/BEN may facilitate CD4+ T-cell recovery and potentially influence post-transplant outcomes. However, in our small cohort, no differences in OS or NRM were observed between the two groups. Moreover, although we were unable to directly analyze the T-cell content in the bone marrow grafts, the composition of mature T cells and their memory distribution in the graft product may represent critical factors influencing the kinetics of early CD4⁺ T-cell recovery in PT-CY/BEN patients following haplo-BMT. Future studies should investigate the potential impact of graft T-cell content on this process.

The reconstitution of NK-cells and γδ T-cells is critical for early immune recovery after haplo-BMT. Both NK-cells and γδ T-cells possess potent cytotoxic capabilities and can mediate GvL effects without relying on major histocompatibility complex (MHC) recognition, reducing the risk of GvHD (53–55). NK-cells rapidly recover post-transplant and provide crucial defense against viral infections and residual malignant cells during the period of adaptive immune reconstitution (56, 57). Similarly, γδ T-cells contribute to infection control and tumor surveillance through their ability to recognize non-peptide antigens and respond to stress-induced ligands on transformed cells and expansion of donor-derived γδ T-cells strongly correlated with event-free and OS in T-cell-replete HSCT (58, 59). PT-CY has been shown to eliminate most mature alloreactive donor NK-cells, blunting NK-cell reconstitution for up to 30 days and creating a window for opportunistic infections and increased risk of relapse (60). Moreover, the immature NK-cells that reconstitute early after PT-CY have been shown to be functionally deficient (61). Interestingly, we observed a trend toward preserved NK-cell numbers in the PT-CY/BEN group (*P=0.09*) one-month post-transplant, particularly among PT-CY/BEN patients that received higher doses of PT-BEN and consequently lower CY. By two months post-transplant, NK-cell counts in PT-CY patients had recovered and remained significantly elevated from day +100 to one year. This elevation in NK-cell counts in PT-CY patients may be attributed to CMV reactivation, which occurred in 60% of PT-CY patients compared to 30% of PT-CY/BEN patients. CMV reactivation has been shown to accelerate NK-cell maturation and promote the expansion of alloreactive NK-cells (62). Regarding γδ T-cells, we did not observe any significant differences between the PT-CY/BEN and PT-CY groups. These findings suggest that PT-BEN/CY may help preserve NK-cell numbers acutely after transplant and potentially reduce the risk of CMV reactivation.

Tregs and MDSCs play pivotal roles in promoting immune tolerance and GvHD in haplo-BMT. Tregs help maintain immune homeostasis by suppressing alloreactive T-cells, thus mitigating immune-mediated tissue damage (63). Similarly, MDSCs contribute to the immunosuppressive microenvironment by inhibiting T-cell activation and promoting donor graft acceptance, enhancing overall transplant outcomes (64). In this Phase 1a/1b trial, we did not observe any effects of PT-BEN/CY on total Treg cell counts or naïve and effector subsets. This Is not surprising, as previous work using murine haplo-BMT models suggests that PT-BEN does not affect early Treg engraftment and mitigates GvHD in the absence of Tregs (16). However, PT-BEN has been shown to promote an increase in MDSC frequency and function in a murine model of haplo-BMT (16). Analogously, we observed a trend towards increased total MDSCs (*P=0.065*) and monocytic MDSC (*P=0.074*) counts two-months after transplant in PT-CY/BEN patients compared PT-CY patients. Monocytic MDSCs have been implicated in the inhibition of effector CD8+ T-cells, which could also further explain the reduced number of CD8+ T-cells in the PT-CY/BEN patients (65, 66). Overall, PT-CY/BEN appears to have minimal effects on immunosuppressive cell reconstitution after transplant, which is supported by the similar GvHD outcomes in both the PT-CY/BEN and PT-CY cohorts.

TCR-β diversity is a key determinant of immune reconstitution following haplo-BMT, playing a crucial role in infection control and relapse prevention (67, 68). A diverse TCR-β repertoire enhances the ability to recognize and respond to a broad array of pathogens, thereby reducing post-transplant infectious complications (69) Furthermore, increased TCR-β diversity has been associated with improved GvL effects, contributing to lower relapse rates in haplo-BMT recipients. In contrast, limited TCR-β diversity can impair immune recovery, increasing susceptibility to infections and reducing GvHD control (70). As such, strategies to optimize TCR-β diversity, such as tailored conditioning regimens or adoptive T-cell therapies, hold promise for improving haplo-BMT outcomes by achieving a balance between effective immune reconstitution and immune tolerance. Our results indicate that PT-CY/BEN could serve as a strategy to improve T-cell repertoire as it was associated with increased diversity and richness (**Figure 8**), suggesting PT-CY/BEN maintains a higher number of diverse T-cell clones from the donor. It has been documented the PT-CY has been associated with a restricted T-cell repertoire that is likely due to strong depletion of alloreactive T-cells (71). However, longitudinal TCR-seq results from the BMT CTN 1801 study reported constrained TCR-β diversity (Clonality, Slope, Richness, Singletons) in patients with PT-CY, shielding patients from cGVHD while increasing their susceptibility to infectious complications (72). This phenomenon is potentially represented in our trial in that the reduced TCR-β diversity observed in the PT-CY cohort appears to be driven by CMV reactivation (**Figure 9**). In our exploratory analysis, patients with CMV reactivation, regardless off cohort, tend to have a lower TCR-diversity and it is well established that CMV reactivation can restrict the T-cell repertoire (47). Taken together, these results suggest that PT-CY/BEN retains a more diverse T-cell compartment by potentially reducing the incidence of viral reactivations.

In summary, our findings demonstrate that incorporating PT-BEN alongside PT-CY in T-cell replete haplo-BMT offers distinct immunologic advantages while maintaining comparable GvHD outcomes. PT-CY/BEN accelerated CD4+ T-cell recovery, enhanced TCR-β diversity, and preserved early NK-cell numbers, potentially reducing risks associated with delayed immune reconstitution, such as infections and relapse. While PT-CY/BEN suppressed CD8+ T-cell counts, leading to an elevated CD4:CD8 ratio, this effect may contribute to reduced GvHD risk without impairing overall immune recovery. Furthermore, the observed trends toward increased MDSCs and reduced CMV reactivation highlight PT-CY/BEN’s potential to optimize immune reconstitution and improve post-transplant outcomes. These findings underscore the promise of PT-CY/BEN as a novel strategy to balance immune tolerance and effective immune recovery, warranting further evaluation in larger clinical trials.

## Data Availability

The raw data supporting the conclusions of this article will be made available by the authors, without undue reservation.
