# Correction: Impact of post-transplant cyclophosphamide with bendamustine on immune reconstitution in young patients undergoing T-cell replete haploidentical bone marrow transplantation: results from a phase Ia/Ib clinical trial

**DOI:** 10.3389/fimmu.2025.1633590

**Published:** 2025-06-26

**Authors:** Forrest L. Baker, Jessica Stokes, Megan J. Cracchiolo, Dan Davini, Richard J. Simpson, Emmanuel Katsanis

**Affiliations:** ^1^ School of Nutritional Sciences and Wellness, University of Arizona, Tucson, AZ, United States; ^2^ Department of Pediatrics, University of Arizona, Tucson, AZ, United States; ^3^ The University of Arizona Cancer Center, Tucson, AZ, United States; ^4^ Department of Immunobiology, University of Arizona, Tucson, AZ, United States; ^5^ Banner University Medical Center, Tucson, AZ, United States

**Keywords:** bendamustine, cyclophosphamide, hematopoietic cell transplantation, TCR-β sequencing, T-cells, cytomegalovirus

In the published article, **Supplementary Table 1** was mistakenly not included in the publication. The missing material appears below:

The authors apologize for this error and state that this does not change the scientific conclusions of the article in any way. The original article has been updated.

