# Supplementary material for: Impact of post-transplant cyclophosphamide with bendamustine on immune reconstitution in young patients undergoing T-cell replete haploidentical bone marrow transplantation: results from a phase Ia/Ib clinical trial
Source: Front Immunol. 2025 Apr 9;16:1568862. doi: 10.3389/fimmu.2025.1568862 (PMC12014643; doi:10.3389/fimmu.2025.1568862)
Supplement: Supplementary file 1 [file Table1.docx]

| T-Cell Panel | CD45 (2D1) | CD45RA (HI100) | CCR7 (3D12) | TCR-γδ (11F2) | CD95 (DX2) | CD31 (WM59) | CD4 (OKT4) | CD3 (UCHT1) | CD8 (SK1) |
| --- | --- | --- | --- | --- | --- | --- | --- | --- | --- |
|  | SuperBright 780 | Pacific Blue | BB515 | Pacific Orange | PE | PE-CF594 | PE-Cy7 | AlexFluor 700 | APC-H7 |
| Treg Panel | CD45 (2D1) | CD45RA (HI100) | CD25 (BC96) | CD127 (HIL-7R-M21) |  |  | CD4 (OKT4) | FoxP3 (236A/E7) |  |
|  | SuperBright 780 | Pacific Blue | BB515 | BV510 |  |  | PE-Cy7 | APC |  |
| NK-Cell Panel | CD45 (2D1) | CD14 (MθP9) | CD56 (B159) |  | CD1d (51.1) |  | CD16 (CB16) | CD3 (UCHT1) |  |
|  | SuperBright 780 | BV421 | FITC |  | PE |  | PE-Cy7 | AlexFluor 700 |  |
| B-Cell Panel | CD45 (2D1) | CD19 (HIB19) | CD24 (ML5) |  |  | CD38 (HIT2) |  | CD27 (M-T271) |  |
|  | SuperBright 780 | BV421 | BB515 |  |  | PE-CF594 |  | APC |  |
| Myeloid Panel | CD45 (2D1) | CD14 (MθP9) | CD16 (3G8) |  | HLA DR (L243) | CD15 (HI98) | CD33 (P67.6) | CD11b (ICRF44) |  |
|  | SuperBright 780 | BV421 | AlexaFluor 488 |  | PE | PerCP-eFlour 710 | PE-Cy7 | APC |  |
| DC Panel | CD45 (2D1) | CD141 (M80) | CD11c (Bu15) |  | CD370 (8F9) | CD135 (BV10A4H2) |  | Lineage |  |
|  | SuperBright 780 | BV421 | AlexaFluor 488 |  | PE | PE/Dazzle 594 |  | APC |  |

**Supplementary Table 1: Antibody Panels**

Antibodies (clone) used were anti-human and purchased from either:

**Invitrogen (ThermoFisher, Carlsbad, CA)**: CD45 (2D1); CD95 (DX2); CD1d (51.1); HLA-DR (L243); CD15 (HI98); CD16 (CB16); FoxP3 (236A/E7); CD11b (ICRF44). **BioLegend (San Diego, CA)**: CD45RA (HI100); CD141 (M80); CD16 (3G8); CD11c (Bu15); CD370 (8F9); CD135 (BV10A4H2); CD4 (OKT4); Lineage. **BD Biosciences (San Jose, CA)**: CD14 (MθP9); CD19 (HIB19); CCR7 (3D12); CD25 (BC96); CD56 (B159); CD24 (ML5); TCR-γδ (11F2); CD127 (HIL-7R-M21); CD31 (WM59); CD38 (HIT2); CD33 (P67.6); CD3 (UCHT1); CD27 (M-T271); CD8 (SK1).
